# *Anopheles sinensis* mosquito insecticide resistance: comparison of three mosquito sample collection and preparation methods and mosquito age in resistance measurements

**DOI:** 10.1186/1756-3305-7-54

**Published:** 2014-01-28

**Authors:** Tielong Xu, Daibin Zhong, Linhua Tang, Xuelian Chang, Fengyang Fu, Guiyun Yan, Bin Zheng

**Affiliations:** 1 National Institute of Parasitic Diseases, Chinese Center for Disease Control and Prevention, and WHO Collaborating Center for Malaria, Schistosomiasis and Filariasis, Ministry of Public Health, Shanghai, China; 2 Program in Public Health, College of Health Sciences, University of California at Irvine, Irvine, CA 92697, USA; 3 Department of Pathogen Biology, Bengbu Medical College, Anhui, China; 4 Institute of Entomology and Molecular Biology, College of Life Sciences, Chongqing Normal University, Chongqing, China

**Keywords:** *Anopheles sinensis*, Pyrethroid resistance, Mosquito preparation methods

## Abstract

**Background:**

Insecticide resistance monitoring in malaria mosquitoes is essential for guiding the rational use of insecticides in vector control programs. Resistance bioassay is the first step for insecticide monitoring and it lays an important foundation for molecular examination of resistance mechanisms. In the literature, various mosquito sample collection and preparation methods have been used, but how mosquito sample collection and preparation methods affect insecticide susceptibility bioassay results is largely unknown. The objectives of this study were to determine whether mosquito sample collection and preparation methods affected bioassay results, which may cause incorrect classification of mosquito resistance status.

**Methods:**

The study was conducted in *Anopheles sinensis* mosquitoes in two study sites in central China. Three mosquito sample collection and preparation methods were compared for insecticide susceptibility, *kdr* frequencies and metabolic enzyme activities: 1) adult mosquitoes collected from the field; 2) F1 adults from field collected, blood-fed mosquitoes; and 3) adult mosquitoes reared from field collected larvae.

**Results:**

Mosquito sample collection and preparation methods significantly affected mortality rates in the standard WHO tube resistance bioassay. Mortality rate of field-collected female adults was 10-15% higher than in mosquitoes reared from field-collected larvae and F1 adults from field collected blood-fed females. This pattern was consistent in mosquitoes from the two study sites. High *kdr* mutation frequency (85-95%) with L1014F allele as the predominant mutation was found in our study populations. Field-collected female adults consistently exhibited the highest monooxygenase and GST activities. The higher mortality rate observed in the field-collected female mosquitoes may have been caused by a mixture of mosquitoes of different ages, as older mosquitoes were more susceptible to deltamethrin than younger mosquitoes.

**Conclusions:**

Female adults reared from field-collected larvae in resistance bioassays are recommended to minimize the effect of confounding factors such as mosquito age and blood feeding status so that more reliable and reproducible mortality may be obtained.

## Background

Malaria is a main cause of morbidity and mortality worldwide. One important tool to prevent and control malaria is vector control, especially using long-lasting insecticide-treated bed nets (LLINs) and indoor residual spraying (IRS) [1]. Currently, WHO recommends pyrethroids for bed-net impregnation and for indoor residual sprays because of their low toxicity to mammals and humans and high efficacy against mosquitoes [2]. The insecticides used in these public health programs have posed strong selection pressure for resistance. The use of insecticides for agricultural purposes also exerts selection pressure for resistance in mosquitoes because mosquito larvae breed in agricultural fields and thus are directly exposed to insecticides. Furthermore, residual insecticides from agricultural pest control may be leaked into mosquito breeding sites and expose mosquito larvae to insecticides. A key element of resistance management is resistance surveillance.

Resistance bioassay is the first important step in insecticide resistance surveillance. Three sources of mosquitoes have been used for bioassays in the literature: 1) adult mosquitoes collected from the fields were directly used for bioassay, regardless of mosquito age and blood feeding status [3-5]; 2) adult mosquitoes reared from field collected larvae, usually at 3–5 days post emergence [6-8]; and 3) F1 adults from field collected blood-fed mosquitoes, usually at 3–5 days post emergence [9,10]. Different mosquito sampling and preparation methods may yield varying results on the knockdown rate and mortality rates because of differences in physiological status (e.g., age and blood feeding status) and possible genetic sampling bias due to small sample size in field mosquito sampling. On the other hand, WHO classifies insecticide resistance status based on bioassay mortality (resistant if mortality rate is <90%, possible resistance if mortality is between 90-97%, and susceptible if the rate is ≥98%) [11]. Therefore, it is important to evaluate the effects of sampling and mosquito preparation methods on the bioassay results so that resistance classification is appropriately conducted. This information will help in understanding the possible bias in bioassay mortality rate and resistance classification resulting from the mosquito sample collection and preparation methods.

Pyrethroid resistance in *Anopheles* mosquitoes has been studied extensively, primarily in *Anopheles gambiae*, the most important African malaria vector. Two major resistance mechanisms have been recognized. The first is point mutations in the *Para*-type sodium channel gene, the target site of pyrethroids, causing a change in affinity between the insecticide and its binding site and leading to knockdown resistance (*kdr*). The most common mutation conferring *kdr* is a mutation at position 1014 causing a change from leucine to either phenylalanine (L1014F) or serine (L1014S) [12-19]. However, mutations at other positions of the *kdr* gene can further enhance pyrethroid resistance (super *kdr* phenotype) [20,21]. The second mechanism is metabolic detoxification of pyrethroids before they reach their target site by detoxification enzymes, including P450 monooxygenase and glutathione-S-transferases [22-24]. The objective of this study is to determine the effect of mosquito sample collection and preparation methods on resistance bioassays, the *kdr* gene mutation, and metabolic detoxification enzyme activities.

## Methods

### Study sites

**Figure 1:**
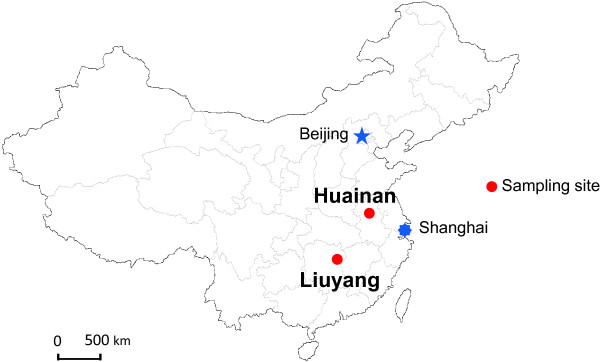
A map of China showing the distribution of mosquito sampling sites.

We conducted the study in two sites in China, Liuyang County (three villages, 28.1 N and 113.4E) in Hunan province and Huainan County (two villages, 32.6 N and 117.1E) in Anhui province (Figure 1). Malaria in Hunan province was historically endemic, and the latest reported local infection was in 2010. Malaria in the Anhui site is hypo-endemic with sporadic vivax malaria outbreaks [25]. Indoor residual spraying in houses surrounding the malaria index case is the main malaria control measure. Rice is the major agricultural crop in these study sites with one harvesting per year. Due to severe insect pest damage on the rice, insecticide use for pest control has been very intensive, with several rounds of sprays in one growing season. Pyrethroids are commonly used for agricultural pest control, but other insecticides, such as organophosphates and carbamates, are also being used [26].

### Mosquito sample collection and preparation and resistance bioassay

The study in Liuyang County, Hunan province was conducted in July and August 2011, and in July and August 2012 for Huainan County, Anhui province. *An. sinensis* is a predominant vector of malaria in the two study sites. In each study site, three mosquito sample collection and preparation methods were used: 1) adult mosquitoes collected from the fields; 2) F1 adults from field collected, bloodfed mosquitoes; and 3) adult mosquitoes reared from field collected larvae. For the first method, 1,000 female *An. sinensis* were collected from the pig or cow shelters between 18:00 h and 21:00 h using aspirators. After identifying mosquitoes to species morphologically, half of the *An. sinensis* female mosquitoes were randomly selected for the resistance bioassay, using 0.05% deltamethrin test paper in the standard WHO tube assay [11]. For the second method, the remaining field-caught and bloodfed mosquitoes (200 mosquitoes) were transferred to a mosquito cage and allowed to lay eggs. Eggs were hatched and larvae were reared in spring water with Tetramin fish food. Immediately after emergence, male and female mosquitoes were separated, and all adults were fed on 10% sucrose solution. F1 female adults at 3–5 days post emergence were used for the deltamethrin resistance bioassay in the standard WHO tube test [11]. For the third method, adult mosquitoes reared from field-collected larvae, 2,000 larvae from more than 100 larval habitats were collected using the standard 350 ml dippers. The larvae were transported to the local rearing facility and reared to adults, and female adults 3–5 days post emergence were tested for deltamethrin resistance using the standard WHO tube [11]. A laboratory susceptible strain that has been maintained in the insectary of the Chinese Center for Disease Control and Prevention in Shanghai, China, for more than 30 years with no insecticide exposure was used as the reference susceptible strain.

### Insecticide susceptibility bioassay

The female adults from the three sources of mosquitoes were tested for susceptibility to deltamethrin, using the standard WHO tube bioassay with 0.05% deltamethrin test papers [11]. For each sample collection and preparation method, 140 to 240 female mosquitoes were exposed to WHO papers impregnated with deltemethrin according to WHO protocol, with 20 to 25 mosquitoes per tube. For each sample collection and preparation method, 7–12 replicates were used. Paraffin oil-treated papers without insecticide (control paper) were also tested, and 20 to 25 mosquitoes were used. Mosquitoes were exposed for 1 hour. The knockdown time of individual mosquitoes was recorded every 10 minutes, and the time required for 50% knockdown of mosquitoes (KT_50_) was determined by the Probit analysis [27] using SAS software. After 1-hour exposure, mosquitoes were transferred to recovery cups and maintained on 10% sucrose solution for 24 hours and the number of surviving mosquitoes was recorded. All bioassay mosquitoes were tested for metabolic detoxification enzyme activities and a subset of samples were tested for *kdr* gene mutations.

### Effects of mosquito age and blood feeding status on resistance

The main factors that confound insecticide resistance in field-collected mosquitoes are bloodfeeding status and mosquito age. Therefore, we examined the effects of mosquito age and blood feeding status on insecticide resistance and the underlying mechanisms such as metabolic detoxification enzyme activities. To test the effects of mosquito age, larvae were collected from Huainan county, Anhui province, and reared to adults. Three-day old (n = 240) and 20-day old (n = 45) non-blood-fed female adults were bioassayed for resistance to deltamethrin using the standard WHO resistance tube assay described above, and the number of mosquitoes that died after the 24-hour recovery period was recorded. To determine the effects of mosquito blood feeding on resistance, field collected female adults in each site, regardless of blood feeding status, were bioassayed for resistance to deltamethrin. The feeding status of each mosquito was then determined, and the survival status after the 24-hour recovery period was recorded. A mosquito is classified as "blood- fed" if she was fully engorged, and as "non-bloodfed" if the stomach did not contain any blood by visual inspection. A total of 240 non-bloodfed and 80 blood-fed mosquitoes were bioassayed. Thirty mosquitoes were selected randomly from each group for enzyme activity and *kdr* mutation detecting.

### Metabolic enzyme activity assays

We followed the previously published protocol to measure the activity of glutathione s-transferase (GST) and monooxygenase [12,28,29]. Briefly, individual females were homogenized in 200 μl of KPO_4_ buffer (0.25 M, pH 7.2) and then diluted by adding phosphate buffer. The tube was mixed, centrifuged, and the supernatant was used to test GST and monooxygenase. All assays were carried out in duplicate.

### Molecular identification and detection of *kdr* mutation

One leg of each mosquito was used for DNA extraction with the Fast Tissue-to-PCR Kit (Fermentas, CA). Briefly, the mosquito leg was placed at the bottom of a 500 μl Eppendorf tube. A total of 50 μl of tissue lysis solution and 5 μl of protein K solution were added and incubated at 55°C for 20 min, followed by 10 minutes at 95°C. After the incubations, 50 μl of neutralization solution was added and mixed by vortexing. The neutralized tissue was then centrifuged at 14,000 rpm for 10 min. Extract DNA was stored at 4°C or used immediately for PCR. Molecular identifications of *An. sinensis* species were conducted by using species-specific primers targeting amplification of the ITS2 and 28S rDNA regions (D1 and D2) [30]. To determine point mutations of the *kdr* gene at position 1014, we amplified a 325 bp fragment, using the primer pair: *kdr*-F TGCCACTCCGTGTGTTTAGA, and *kdr*-R GAGCGATGATGATCCGAAAT. PCR primers were designed based on the *An. sinensis* sequences of the DIIS6 (domain 2 S6) region of the *para-type* sodium gene (GenBank acc. no. DQ334052). PCR products were directly sequenced using the big-dye kit by Sangon Biotech CO., Ltd. (Shanghai).

### Statistical analysis

The mortality rate of the mosquitoes exposed to test papers was adjusted by the mortality rate of the mosquitoes in the control group (exposed to paraffin oil-treated papers without insecticide), according to Abbott’s formula [11]. To determine the effects of mosquito sample collection and preparation methods, analysis of variance (ANOVA) was conducted using arcsine transformation rate of mosquito mortality from the insecticide susceptibility bioassays and Duncan multiple range tests were used to determine pair-wise differences. One-way ANOVA was also used to examine the difference in monooxygenase and GST activity among the mosquitoes from three sample collection and preparation methods. Statistical significance of differences in mortality between blood-fed and non-bloodfed mosquitoes, or between young (3-day old) and old (20-day old) mosquitoes was examined using the Chi-square test, and the t-test was used to determine the statistical difference in monooxygenase and GST activities. The *kdr* allele frequency was calculated in each site in each sample collection and preparation method, and between susceptible and resistant mosquitoes. Statistical differences among sample collection and preparation methods were examined using ANOVA, and statistical differences between susceptible and resistant mosquitoes were examined using the Chi-square test.

### Ethics statement

No specific permits were required for the described field studies. For mosquito collection in rice paddies, oral consent was obtained from field owners in each location. These locations were not protected land, and the field studies did not involve endangered or protected species.

## Results

### Relationship between mosquito sample collection and preparation methods and bioassay mortality rates

For both the Hunan and Anhui study sites, the mortality rate of field-collected adults was significantly higher than in the adults reared from field-collected larvae and in F1 adults from field-collected blood-fed females (Table 1). For example, in the Anhui study site, a mortality rate of 47.6% was detected for field-collected adults, significantly higher than the adults reared from field-collected larvae (31.7%) and F1 adults from field collected blood-fed females (32.9%). The significantly higher mortality rate in field-collected adults suggests that these individuals were more susceptible to the insecticides, likely due to a mixture of mosquitoes of different ages and various blood feeding statuses within this group. The KT_50_ varied 92–101 min, and there was no significant difference among the mosquitoes from the three different preparation methods (Table 1). Similar patterns were observed in the Hunan study site (Table 1), although the mortality rates for mosquitoes in all three preparation methods were lower than the Anhui study site, suggesting the Hunan site exhibited higher resistance than the Anhui site. The laboratory susceptible strain showed a mortality rate of 99%. Therefore, both the Hunan and Anhui populations can be classified as "resistant" according to the WHO classification on insecticide resistance [11] because the mortality rates in all three mosquito sample collection and preparation methods were lower than 90%.

**Table 1:** **Comparison of mortality rates and knockdown time among three sources of *Anopheles sinensis *** **mosquitoes using the standard WHO deltamethrin resistance bioassay**

			**Mortality**		**Knockdown time**	
**Site**	**Source of mosquitoes**	**n**	Mean (%)*	**95% confidence interval**	**KT**_**50**_(min)*	**95% confidence interval**
Anhui (Huainan county)	Field-collected female adults	140	47.6^a^	42.4 - 52.8	97^a^	81.2 - 131.5
	Female adults reared from field-collected larvae	240	31.7^b^	28.7 - 34.7	92^a^	72.3 - 155.5
	F1 adults from field blood-fed female	141	32.9^b^	27.9 - 37.9	101^a^	83.9 - 143.9
Hunan (Liuyang county)	Field-collected female adults	180	24.9^a^	19.2 - 30.6	104	83.4 - 170.8
	Female adults reared from field-collected larvae	234	15.5^b^	11.8 - 19.1	—	—
	F1 adults from field blood-fed female	150	15.1^b^	12.6 - 17.6	—	—
	Laboratory strain	125	99.0	98.6 - 99.4	15	14.7 - 16.1

*For each site, the mortality rate or KT_50_that shares different letter indicates statistical difference at P < 0.05 using the Duncan multiple range test.

—No mosquitoes were knocked down within the 60-min exposure time, thus KT_50_ could not be calculated.

### Effects of blood feeding status and mosquito age on bioassay mortality

Blood feeding status had no significant effects on mosquito mortality in the bioassays. The mortality rates of blood-fed and non-bloodfed mosquitoes were 35.4% and 38.8%, respectively (*χ*^*2*^ = 0.45, df = 1; *P*> 0.05) (Table 2). However, mosquito age showed a significant effect, with significantly higher mortality in the 20-day old mosquitoes (55.7%) than in the 3-day old mosquitoes (31.7%; *χ*^*2*^ = 9.46, df = 1; *P*< 0.01), suggesting older mosquitoes were more susceptible to deltamethrin insecticide.

**Table 2:** **Effects of blood-feeding status and mosquito age on insecticide resistance bioassay in *Anopheles sinensis *** **mosquitoes**

			**Mortality**		**Knockdown time**	
**Trait**	**Group**	**n**	Mortality rate (%)*	**95% confidence interval**	**KT**_**50**_(min)*	**95% confidence interval**
Blood feeding status	Bloodfed	240	35.4^a^	30.3 - 40.5	—	—
	Non-bloodfed	80	38.8^a^	33.1 - 44.5	—	—
Mosquito age	3-day old	240	31.7^a^	28.7 - 34.7	216^a^	81.4 – 131.5
	20-day old	45	55.6^b^	52.5 - 58.7	106^a^	61.9 - 184.5

*For each trait, the mortality rate that shares different letter indicates statistical difference at P < 0.05 using the chi-square test.

—Not done.

### *Kdr* allele frequencies in *An. sinensis* populations

We genotyped a total of 598 mosquitoes collected from the field, including 451 mosquitoes that survived the bioassay (resistant), 147 dead mosquitoes (susceptible), and 50 from the laboratory susceptible colony (Table 3). Three *kdr* alleles at codon 1014 were detected: 1) TTG (wildtype), 2) TGT which causes non-synonymous mutation from leucine (L) to cysteine (C), and 3) TTT and TTC which causes substitution of leucine (L) by phenylalanine (F). We calculated *kdr* allele frequency for mosquitoes from the three different sample collection and preparation methods for each site (Table 3). Overall, the individuals that we analyzed for *kdr* in the Hunan and Anhui populations exhibited very high *kdr* mutation frequencies, with the L1014F and L1014C mutation frequencies exceeding 85%, and the L1014F mutation was the dominant mutation. This is consistent with the previous findings on high *kdr* mutation frequencies in these study sites [31]. In contrast, the laboratory susceptible population showed very low *kdr* mutation frequency (1%). Secondly, the mosquitoes that survived the bioassay (i.e., resistant individuals) consistently showed higher frequencies of mutated *kdr* alleles than those that died in the bioassay (i.e., susceptible individuals) (Table 3), suggesting *kdr* mutation played a small role in resistance. Overall, the mutated *kdr* allele frequency in the resistant individuals was 92.1%, significantly higher than the susceptible individuals (84.0%) (*χ*^*2*^ = 20.4, df = 1; *P*< 0.001).

**Table 3:** ***Kdr *** **allele frequency (in percentage) of *Anopheles sinensis *** **mosquitoes among the three sources of mosquitoes used in the bioassay**

**Study**	**Sample preparation**	**Bioassay**		**L1014F**	**L1014C**	**Wildtype L1014**	Population mutation frequency*
**Site**	**Methods**	**Status**	**n**	**(TTT + TTC)**	**(TGT)**	**(TTG)**	**(TTT+TTC+TGT)**
Anhui (Huainan county)	Female mosquitoes directly collected from the field	Alive	44	81.8	14.8	3.4	94.5^a^
		Dead	37	63.5	28.4	8.1	
	Female mosquitoes reared from field-collected larvae	Alive	55	78.2	19.1	2.7	94.4^a^
		Dead	26	61.5	26.9	11.5	
	F1 female from field collected bloodfed females	Alive	53	83.0	11.3	5.7	91.2^b^
		Dead	27	66.7	18.5	14.8	
Hunan (Liuyang county)	Female mosquitoes directly	Alive	89	79.2	8.4	12.4	84.7^a^
	Collected from the field	Dead	16	62.5	6.3	31.3	
	Female mosquitoes reared from field-collected larvae	Alive	121	78.5	12.0	9.5	88.4^b^
		Dead	25	64.0	14.0	22.0	
	F1 female from field collected bloodfed females	Alive	89	74.2	17.9	7.9	90.5^b^
		Dead	16	56.3	25.0	19.7	
Total		Alive	451	78.6	13.5	7.9	
		Dead	147	62.9	21.1	16.0	
Laboratory strain		Dead	50	1.0	0.0	99.0	1.0

*For each site, the populations that share different letters indicate statistical difference at P < 0.05 using the analysis of molecular variance.

### Metabolic enzyme activities

**Figure 2:**
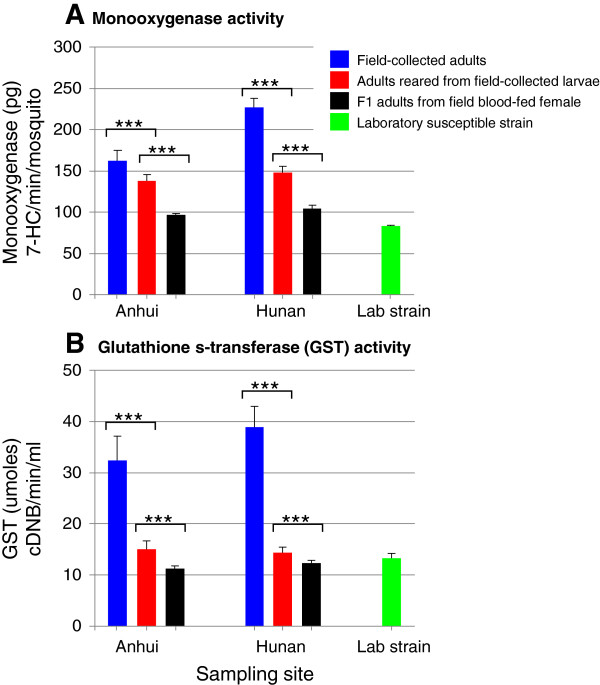
**Effects of sample collection and preparation methods on metabolic detoxification enzyme activities in *Anopheles sinensis *mosquitoes. A**: P450 monooxygenases; and **B**: glutathione S-transferases. ***, P < 0.001.

**Figure 3:**
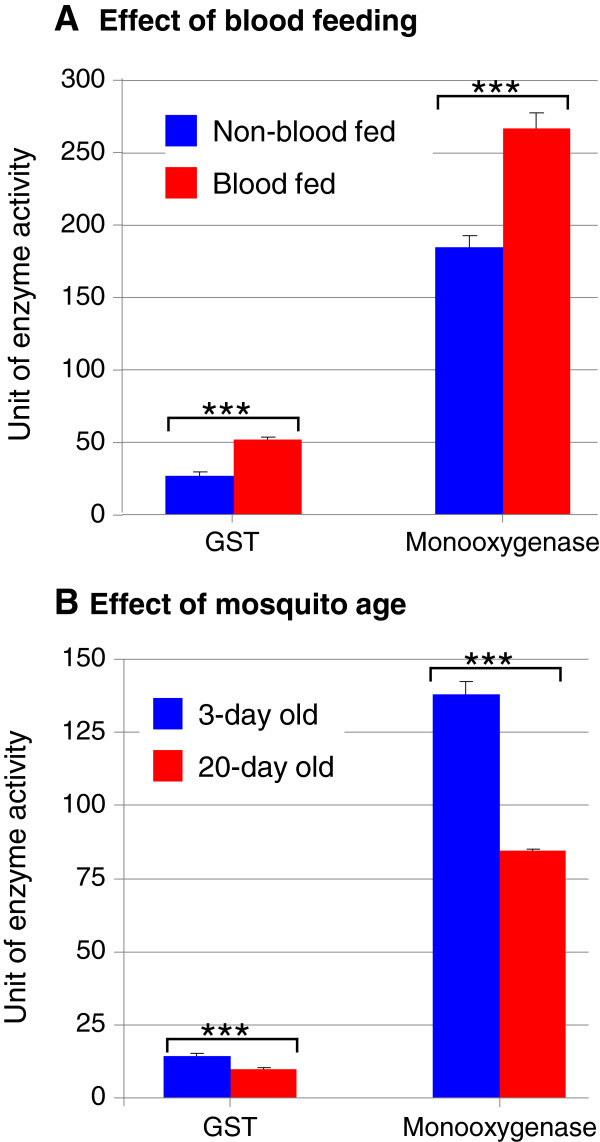
**Effects of bloodfeeding (A) and mosquito age (B) on metabolic detoxification enzyme activities in *Anopheles sinensis *mosquitoes.** ***, P < 0.001.

We measured P450 monooxygenase and glutathione s-transferase (GST) activities for mosquitoes from three sample collection and preparation methods for each site. In both study sites, field-collected female adults consistently exhibited the highest monooxygenase and GST activities, nearly two fold higher than F1 adults from field blood-fed females for monooxygenase (Figure 2A) and three fold higher for GST (Figure 2B). Bloodfeeding increased GST activity by 95.1% (t = 6.54, df = 1, P < 0.001) and increased monooxygenase activity by 44.3% (t = 6.82, df = 1, P < 0.001) (Figure 3A). The 20-day old female mosquitoes exhibited significantly lower GST activity (t = 6.53, df = 1, P < 0.001) and lower monooxygenase activity (t = 12.28, df = 1, P < 0.001) than the 3-day old female mosquitoes (Figure 3B).

## Discussion

In 2010 China revised its national malaria control strategy and set the goal of malaria elimination by 2020 [32]. Malaria vector control is a key component of the malaria control strategy. However, the rapid rise and spread of insecticide resistance have become major threats to the efficiency of insecticide-based vector control activities. Insecticide resistance monitoring in *Anopheles* mosquitoes is essential to guide the rational use of insecticides in vector control programs. Resistance bioassay with WHO standard tubes or bottles is the first step to quantify insecticide resistance in mosquito populations. At least three mosquito sample collection and preparation methods have been used in the literature, including field-collected adult mosquitoes, F1 adults from blood-fed females collected from the field, and F1 female adults from field-collected larvae as recommended by WHO [11]. Un-resolved issues include: 1) whether mosquito sample collection and preparation methods affect bioassay results, and 2) how much the other sample collection and preparation methods lead to a biased estimation of mosquito mortality rate and thus incorrectly classify the population resistance status. This study was designed to address these two questions using two independent study sites.

We found that mosquito sample collection and preparation methods significantly affected mortality rates in the standard WHO tube resistance bioassay. In particular, the mortality rate of field-collected female adults was the highest, 10-15% higher than mosquitoes reared from field collected larvae and F1 adults from field blood-fed females. On the other hand, mosquitoes reared from field collected larvae and F1 adults from field blood-fed females exhibited similar mortality rates. This pattern was consistent between the two study sites. Therefore, we conclude that when comparing insecticide resistance across multiple study sites, the same mosquito sample collection and preparation method must be used, particularly when field-collected female mosquitoes are directly used in the bioassay. Because our study populations are highly resistant to deltamethrin, the higher mortality rate detected in field-collected female mosquitoes did not lead to incorrect resistance classification based on the WHO standard [11]. However, the 10-15% higher mortality rate would become critical when mortality rate of the mosquito population is near the threshold level (i.e., 90% mortality) for resistance classification.

The higher mortality rate observed in the field-collected female mosquitoes is likely to be a result of variability in age. Field-collected female mosquitoes represent a mixture of mosquitoes of different ages and different blood-feeding history, which may confound the bioassay results [22,33-35]. In this study we found that older mosquitoes were far more susceptible to deltamethrin than younger mosquitoes. We did not detect significant effects of bloodfeeding on bioassay mortality in this study. Perhaps non-blood-fed mosquitoes may be more active in searching for a bloodmeal and may have a higher chance of being exposed to insecticide-treated nets in natural conditions. However, under our bioassay conditions all mosquitoes were confined to small tubes and all would have a high chance of exposure to the deltamethrin test paper, which may explain why we did not detect significant effects of bloodfeeding on mortality in the susceptibility bioassay.

It is interesting to note that field-collected mosquitoes exhibited the highest monooxygenase and GST activities - several folds higher than F1 adults from blood-fed females or female adults reared from field-collected larvae. Such high levels of monooxygenase and GST activities may also be partially caused by the bloodfeeding event because most field-collected females were blood-fed and blood feeding increased GST activity by 95% and monooxygenase activity by 44%. A recent study that used the stepwise multiple regression analyses in *An. sinensis* mosquito populations from central and southern China that demonstrated both *kdr* mutations and monooxygenase activity were significantly associated with deltamethrin resistance, with monooxygenase activity playing a stronger role [31].

## Conclusions

Considering that the field-collected adults could not eliminate the confounding effects of blood feeding and mosquito age and this could likely lead to a biased estimate of insecticide resistance, F1 female adults reared from field-collected larvae should be used as the first line technique in resistance bioassays because it minimizes the effect of confounding factors such as mosquito age and physiological status so that more reliable and reproducible mortality will be obtained. We do not recommend using field-collected adults in bioassays during resistance monitoring because mixed blood feeding status and mosquito ages in the test specimens likely leads to less reliable and less reproducible mortality estimates. F1 adults from blood-fed females collected from the field may be used for bioassay only when the first line technique is unattainable and when F1 adults are from a large number of field-collected blood-fed females and the founder effect is minimized.

## Competing interests

The authors declare that they have no competing interests.

## Authors’ contributions

Conceived and designed the experiments: BZ and GY. Performed the experiments: TX, DZ, XC and FF. Analyzed the data: TX. Wrote and revised the manuscript: TX, LT, BZ and GY. All authors read and approved the final manuscript.
